# Multimodality Imaging and Biomarker Approach to Characterize the Pathophysiology of Heart Failure in Left Ventricular Non-Compaction with Preserved Ejection Fraction

**DOI:** 10.3390/jcm12113632

**Published:** 2023-05-23

**Authors:** Ionela-Simona Visoiu, Roxana Cristina Rimbas, Alina Ioana Nicula, Sorina Mihaila-Baldea, Stefania Lucia Magda, Diana Janina Mihalcea, Memis Hayat, Maria Luiza Luchian, Alexandra Maria Chitroceanu, Dragos Vinereanu

**Affiliations:** 1 Department of Cardiology and Cardiovascular Surgery, University of Medicine and Pharmacy Carol Davila, 37 Dionisie Lupu, 020021 Bucharest, Romania; simona.visoiu@drd.umfcd.ro (I.-S.V.); roxana.rimbas@umfcd.ro (R.C.R.); alinapavel74@yahoo.com (A.I.N.); sorina.mihaila@umfcd.ro (S.M.-B.); stefania.magda@umfcd.ro (S.L.M.); dyddy_free@yahoo.com (D.J.M.); hayat_memis@yahoo.com (M.H.); 2 Department of Cardiology, University and Emergency Hospital, 169 Splaiul Independentei, 050098 Bucharest, Romania; marialuiza.luchian@yahoo.com (M.L.L.); alexandra.chitroceanu@yahoo.com (A.M.C.); 3 Department of Radiology, University and Emergency Hospital, 169 Splaiul Independentei, 050098 Bucharest, Romania

**Keywords:** left ventricular non-compaction, heart failure with preserved ejection fraction, speckle tracking echocardiography, cardiac magnetic resonance, fibrosis, Galectin-3, endothelial dysfunction

## Abstract

Left ventricular non-compaction (LVNC) with preserved ejection fraction (EF) is still a controverted entity. We aimed to characterize structural and functional changes in LVNC with heart failure with preserved EF (HFpEF). Methods: We enrolled 21 patients with LVNC and HFpEF and 21 HFpEF controls. For all patients, we performed CMR, speckle tracking echocardiography (STE), and biomarker assessment for HFpEF (NT-proBNP), for myocardial fibrosis (Galectin-3), and for endothelial dysfunction [ADAMTS13, von Willebrand factor, and their ratio]. By CMR, we assessed native T1 and extracellular volume (ECV) for each LV level (basal, mid, and apical). By STE, we assessed longitudinal strain (LS), globally and at each LV level, base-to-apex gradient, LS layer by layer, from epicardium to endocardium, and transmural deformation gradient. Results: In the LVNC group, mean NC/C ratio was 2.9 ± 0.4 and the percentage of NC myocardium mass was 24.4 ± 8.7%. LVNC patients, by comparison with controls, had higher apical native T1 (1061 ± 72 vs. 1008 ± 40 ms), diffusely increased ECV (27.2 ± 2.9 vs. 24.4 ± 2.5%), with higher values at the apical level (29.6 ± 3.8 vs. 25.2 ± 2.8%) (all *p* < 0.01); they had a lower LS only at the apical level (−21.4 ± 4.4 vs. −24.3 ± 3.2%), with decreased base-to-apex gradient (3.8 ± 4.7 vs. 6.9 ± 3.4%) and transmural deformation gradient (3.9 ± 0.8 vs. 4.8 ± 1.0%). LVNC patients had higher NT-proBNP [237 (156–489) vs. 156 (139–257) pg/mL] and Galectin-3 [7.3 (6.0–11.5) vs. 5.6 (4.8–8.3) ng/mL], and lower ADAMTS13 (767.3 ± 335.5 vs. 962.3 ± 253.7 ng/mL) and ADAMTS13/vWF ratio (all *p* < 0.05). Conclusion: LVNC patients with HFpEF have diffuse fibrosis, which is more extensive at the apical level, explaining the decrease in apical deformation and overexpression of Galectin-3. Lower transmural and base-to-apex deformation gradients underpin the sequence of myocardial maturation failure. Endothelial dysfunction, expressed by the lower ADAMTS13 and ADAMTS13/vWF ratio, may play an important role in the mechanism of HFpEF in patients with LVNC.

## 1. Introduction

Left ventricular non-compaction (LVNC) is characterized by the morphologic findings of a bilayer myocardium, with hypertrabeculation of the endocardial layer, a usually thin compacted epicardial layer, and the presence of deep intertrabecular recesses that communicate with the left ventricular (LV) cavity [1]. LVNC carries a high risk of heart failure (HF) [2]. Many studies have been conducted to describe the myocardial deformation pattern of patients with LVNC by speckle tracking echocardiography (STE) or cardiac magnetic resonance (CMR) [3,4,5,6], including all spectrums of LVNC, with both reduced or preserved ejection fraction (EF), with or without HF, and comparing them with healthy volunteers or other cardiomyopathies. However, the pathophysiology of HF in LVNC with preserved EF remains elusive and challenging.

Myocardial compaction gradually progresses from epicardium to endocardium and from base to apex [7,8]. The mature myocardial wall is the product of two relatively heterogeneous myocardial layers of the primitive heart tube undergoing different proliferative processes [9,10]. The endocardial layer, with a higher growth rate in the early stage of embryogenesis, generates trabeculae in order to increase cardiac output and expand the surface area for oxygen and nutrient absorption prior to coronary vascularization [10]. The epicardial layer, as an already compacted region of cardiomyocytes, has a growth rate limited initially by the diffusion of oxygen and nutrients through the trabecular layer. As the cardiomyocytes in the compact layer expand, mature, and differentiate, this layer becomes structurally more complex, requiring more oxygen [10]. Hypoxia stimulates angioblast invasion from the epicardium, leading to coronary vascularization [7]. In parallel with coronary vascularization, by trabeculae coalescence the inter-trabecular recesses are compressed to capillaries, connecting with the epicardial coronary arteries [8,9,10]. The impairment of this complex process of myocardial compaction suggests that LVNC may involve both myocardial and microvascular dysfunction, possibly interrelated, leading to HF. A post-mortem study of hearts with LVNC demonstrated subendocardial ischemic lesions [11]. Further studies demonstrated subendocardial perfusion defects and coronary microvascular dysfunction (CMD), with decreased coronary flow reserves, in the absence of epicardial coronary artery disease [12,13,14,15]. CMD may be linked to endothelial dysfunction, leading to reduced nitric oxide (NO) availability and promoting the proliferation of fibroblasts, with an increased content of extracellular matrix proteins. Consequently, the distance of oxygen diffusion between capillaries and myocytes increases, exposing the myocardium to the risk of hypoxia under conditions of reduced blood flow, developing a vicious loop [16]. In line with this hypothesis, LV endomyocardial biopsy in patients with LVNC showed myocardial fibrosis, while CMR showed extracellular volume (ECV) expansion by diffuse fibrosis [17,18].

We aimed to characterize structural and functional changes in patients with LVNC and HF with preserved ejection fraction (HFpEF), by comparison with patients with HFpEF but without LVNC, by using cardiac magnetic resonance (CMR), echocardiography with speckle tracking, and biomarkers.

## 2. Materials and Methods

### 2.1. Study Population

In this prospective, single-center study, we enrolled 21 ambulatory patients over 18 years of age and in sinus rhythm, who fulfilled diagnostic criteria for LVNC and HFpEF [19]. We defined LVNC using the Stöllberger echocardiographic criteria [19], which implies the presence of >3 prominent trabeculations along the left ventricular endocardial border, visible in end-diastole, distinct from papillary muscles, false tendons, or aberrant bands, moving synchronously with the compact (C) myocardium. These trabeculations form the non-compact (NC) part of a two-layered myocardial structure, best visible at end-systole, and the perfusion of the intertrabecular spaces from the ventricular cavity is present at end-diastole using color-Doppler echocardiography [19]. The LVNC diagnosis was further confirmed by CMR using Petersen criteria [20] (NC/C ratio > 2.3 in end-diastole in long axis images) and/or Jacquier criteria [21] (NC mass > 20% of the LV mass).

HFpEF was defined according to the current guidelines’ diagnostic criteria [22]: presence of symptoms and signs of HF, left ventricular ejection fraction (LVEF) > 50% and objective evidence of cardiac structural [LV mass index (LVMI) > 95 g/m^2^ in females and >115 g/m^2^ in males, relative wall thickness (RWT) > 0.42, left atrial volume index (LAVI) > 34 mL/m^2^)] and/or functional abnormalities [E/e’ ratio at rest > 9, pulmonary artery systolic pressure (PASP) > 35 mmHg] consistent with the presence of LV diastolic dysfunction/raised LV filling pressures, including raised natriuretic peptides [N-terminal pro-B-type natriuretic peptide (NT-proBNP) > 125 pg/mL). We used as a control group 21 patients with similar age and sex, with diagnostic criteria for HFpEF but no echocardiographic and CMR criteria for LVNC. For all patients, we performed clinical examination, 12-lead electrocardiogram, and 2D echocardiography (2DE) with STE, CMR, and blood tests. Exclusion criteria were: recent hospitalization (<4 weeks) for acute HF, acute or chronic coronary syndromes, pericardial diseases, previous history of myocarditis, significant valvular heart diseases, hypertrophic cardiomyopathy, Fabry disease, amyloidosis, renal failure with hemodialysis, moderate or severe anemia, sustained atrial arrhythmias, inappropriate quality of echocardiographic images for STE analysis, and contraindication for CMR. Informed consent was obtained in all subjects prior to enrolment. The study protocol conformed to the ethical guidelines of the 1975 Declaration of Helsinki and was approved by the Ethics Committee of the University and Emergency Hospital (Bucharest, Romania).

### 2.2. Cardiac Magnetic Resonance

CMR was performed using a 1.5-T MR scanner (Magnetom Sempra, Siemens Healthcare GmnH, Erlanger, Germany). CMR analysis was performed by a single cardiac expert radiologist (A.I.N), using the syngo.MR Cardiology VB20A post-processing software (syngo.via, Siemens Healthcare GmnH, Erlanger, Germany). Steady-state free precession cine images and late gadolinium enhancement (LGE) images were acquired according to the current recommendations [23]. Left ventricular end-diastolic volume index (LVEDVi), left ventricular end-systolic volume index (LVESVi), and LVEF were measured according to recommendations [23]. T1 mapping was performed using an ECG-triggered single-shot Modified Look-Locker Inversion recovery (MOLLI) sequence, as previously described [24]. Three MOLLI short-axis images (basal, mid, and apical slices) were acquired prior to (native) and 15 min after an intravenous bolus of 0.2 mmol/kg of gadolinium-based contrast (ClariscanTM, GE Healthcare AS, Oslo, Norway). Care was taken to avoid contamination with signals from the blood pool. Myocardial T1 images were segmented as per the American Heart Association 17-segment model. The apex (segment 17) is usually extremely thin in LVNC and was excluded from the analysis. ECV was calculated using the partition coefficient (ʎ) and contemporaneous hematocrit (HCT) [25]. After T1 and ECV measurements, the following parameters were calculated:

- mean value of native T1 for all segments (T1 global), for basal (T1 basal), mid (T1 mid), and apical (T1 apical) segments, and the gradient between apical and basal T1 (T1 base-to-apex gradient);
- mean value of ECV for all segments (ECV global), for basal (ECV basal), mid (ECV mid) and apical (ECV apical) segments, and the gradient between apical and basal ECV (ECV base-to-apex gradient).

### 2.3. Echocardiography

Conventional transthoracic echocardiography (TTE) was performed using a commercially available ultrasound system (Vivid E9, GE Vingmed Ultrasound AS, Horten, Norway) with a 3.5 MHz transducer. Standard images were acquired and digitally stored for offline analysis using a vendor-specific software (EchoPAC, version 113, GE Healthcare, Horten, Norway). The quantification of the cardiac chamber size and function was performed based on the current guidelines’ recommendations [26]. LVEF was calculated according to the modified Simpson’s rule. LVMI was calculated using the linear method from the parasternal long-axis view (the cube formula), indexed to the body surface area (BSA) [26]. LAVI was calculated using the biplane disk summation technique and indexed to the BSA [26]. Tissue Doppler velocities of the septal and lateral mitral annulus were recorded, and their mean was calculated (e’). Early diastolic mitral inflow peak velocity (E wave) was measured, and the E/e’ ratio was calculated. Tricuspid regurgitant jet velocity and inferior vena cava diameter were measured for the estimation of the PASP.

**Figure 1:**
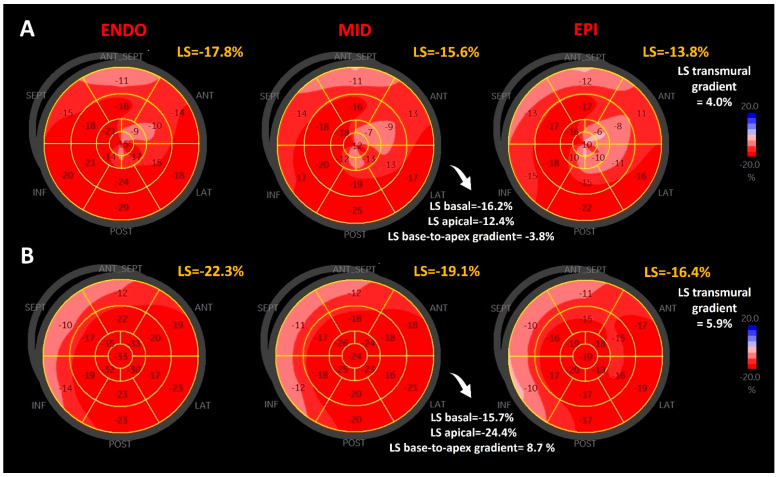
Comparison of LS gradients between a patient with LVNC and a patient without LVNC (control). Bull’s eye plots of LS for the endocardial layer (ENDO), mid-layer (MID), and epicardial layer (EPI) in a 65-year-old female with LVNC (panel **A**) and a 65-year-old male without LVNC (panel **B**), showing lower base-to-apex and transmural deformation gradients in LVNC; LVNC, left ventricular non-compaction; LS, longitudinal strain.

STE analysis was performed by two observers (I.S.V., R.C.R.) according to the current recommendations [26]. The echocardiographic protocol included three apical views (four-, two-, and three-chamber), optimized to avoid foreshortening. For each view, three consecutive heart cycles were recorded during breath-hold, at a similar heart rate, with a frame rate ranging between 60 and 90 frames/s. The endocardial border was manually traced and width of the region of interest (ROI) was adjusted according to the myocardial wall thickness. For LVNC patients, in the non-compacted segments we tracked only the compact myocardial layer, using its inner border as a pseudo-endocardial border. For each ventricular view, the software divided the walls into three separate layers of the myocardial wall: endocardial, mid-myocardial, and epicardial. Before processing and validating the strain curves, the integrity of the myocardial tracking was visually confirmed for each segment of the ROI. Segments with inadequate tracking were excluded from analysis. Subjects with more than one rejected segment per view were excluded from the study. Once all the three apical images were interrogated, the software generated the bull’s eye plot of the 17 LV segments per layer, according to the American Heart Association model (Figure 1). The average of the 17 segments of the mid-myocardial layer bull’s eye plot was defined as the global longitudinal strain (LS). Regional strain analysis was based on the strain value for each segment of this plot. Based on these 17 segments strain measurements, we calculated an average of LS for the basal (LS basal), mid (LS mid), and apical (LS apical) LV levels. The LS base-to-apex gradient was calculated as the difference between apical and basal LS (Figure 1). The average of the 17 segments of endocardial and epicardial layer bull’s eye plots, respectively, were defined as LS endo and LS epi. The transmural LS gradient was calculated as the difference between LS endo and LS epi (Figure 1).

### 2.4. Biomarkers

Blood samples were collected into syringes pre-loaded with EDTA. Two blood test tubes were obtained from each patient. They were immediately centrifuged for 15 min at 4000 rpms to separate serum (2.5–3 mL of serum/patient). Serum was stored at −80 °C before being analyzed for the measurement of specific biomarkers for HF (NT-proBNP), myocardial fibrosis (Galectin-3), and endothelial dysfunction (vWF-von Willebrand factor, ADAMTS13-a disintegrin and metalloproteinase with thrombospondin type 1 motif-13). Plasma vWF, ADAMTS13, and Galectin-3 levels were determined using the sandwich ELISA method with available commercial kits, according to the manufacturer’s instructions.

### 2.5. Statistical Analysis

All statistical analyses were performed using the SPSS version 21.0 (IBM Corp., Armonk, NY, USA). The normal distribution of the continuous variables was tested by the Shapiro–Wilk test. Normally distributed continuous variables were reported as mean ± SD and compared for statistical significance using independent sample *t*-tests. Non-normally distributed continuous variables were presented as median and interquartile range (IQR) and compared using the Mann–Whitney U test. Categorical variables were expressed as percentages and compared using Pearson’s Chi-square test. Correlation between continuous variables was performed using Pearson’s or Spearman’s correlation coefficient, as appropriate. A *p*-value < 0.05 was considered statistically significant.

## 3. Results

### 3.1. Characteristics of the Study Groups

We screened 47 patients potentially having LVNC, with 26 patients being excluded: 5 patients with <4 prominent trabeculations along the LV endocardial border, 6 patients without HF, 11 patients with LVEF < 50%, 2 patients with poor image quality, 1 patient with severe aortic regurgitation, and 1 patient who refused to undergo CMR. The characteristics of the study groups are summarized in Table 1. In the LVNC group, based on CMR, the NC/C ratio was 2.9 ± 0.4 and percent of NC myocardium mass was 24.4 ± 8.7%. Age, gender, BMI, heart rate, systolic blood pressure, and comorbidities were similar between groups. LVEDVi and LVESVi were mildly increased in the LVNC group, whereas LVEF was similar, assessed either by CMR or TTE. LS, structural parameters, and parameters of LV diastolic function were similar between groups.

### 3.2. Cardiac Magnetic Resonance

**Figure 2:**
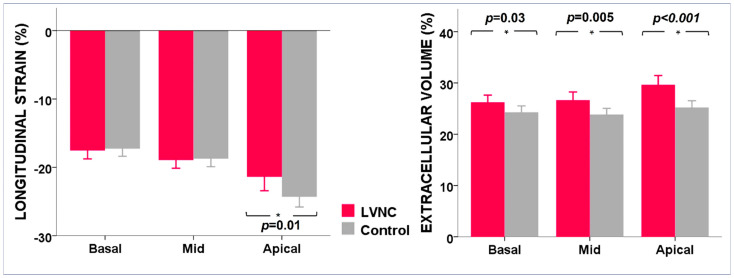
Comparison between LVNC group (in pink) and the control group (in grey) by LV levels, using STE and CMR. Bar charts show averages of longitudinal strain (**left** panel) and extracellular volume (**right** panel) in the basal, mid, and apical LV levels. Error bars represent SD; LVNC, left ventricular non-compaction; LV, left ventricle.

**Figure 3:**
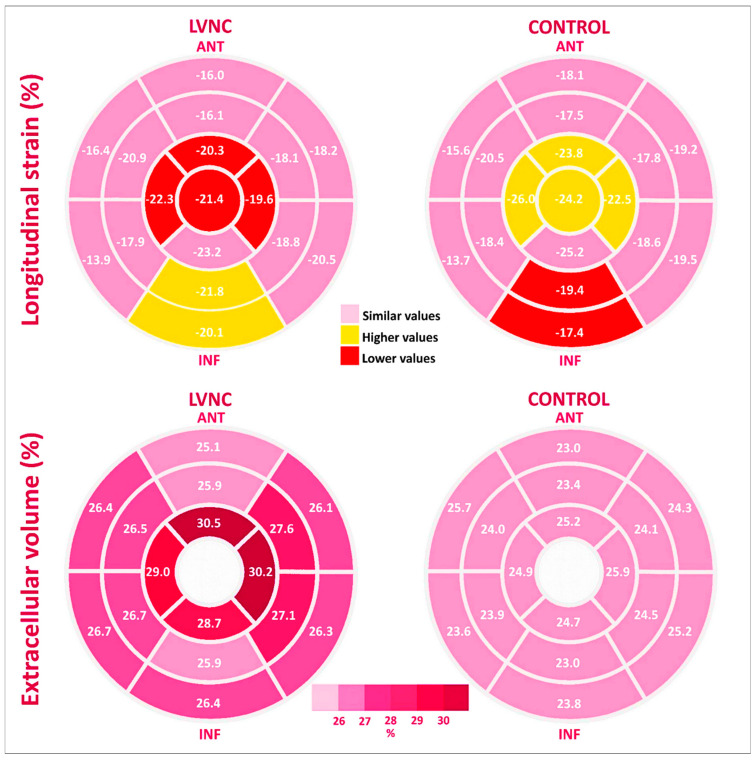
Comparison between LVNC group and the control group by myocardial segments, using STE and CMR. Bull’s-eye plots show averages for longitudinal strain (**upper** panel) and extracellular volume (**lower** panel), for each segment.

In the LVNC group, native T1 values were significantly higher only at the apical level, whereas ECV was globally expanded, mainly at the apical level (Table 2, and Figure 2 and Figure 3). Both T1 base-to-apex gradient and ECV base-to-apex gradient were significantly higher in the LVNC group. Presence of LGE was not different between the two groups (*p* = 0.63), being found in 4 LVNC patients (19%) and in 3 control patients (14%), in both groups in the mid-basal segments.

### 3.3. Regional Strain

LS apical was lower in the LVNC group, by comparison with the control group (Table 3, and Figure 2 and Figure 3), due to significantly lower values of peak LS at the apex and at the apical septal, apical anterior, and apical lateral segments. LS basal and LS mid were not different between groups. LS base-to-apex gradient was also lower in the LVNC group, by comparison with the control group, as was the LS transmural gradient (Table 3).

### 3.4. Biomarkers

In the LVNC patients, NT-proBNP and Galectin-3 were higher, whereas ADAMTS13 and the ADAMTS13/vWF ratio were lower (Table 1).

### 3.5. Correlations

In the LVNC patients, the percent of NC myocardium mass correlated positively with T1 base-to-apex gradient (R = 0.510, *p* = 0.01) and ECV base-to-apex gradient (R = 0.555, *p* = 0.01), while compact myocardium mass correlated negatively with the ECV base-to-apex gradient (R = −0.639, *p* = 0.002). These correlations suggest that fibrosis is higher when the degree of hypertrabeculation increases and the compact myocardium mass decreases.

In the LVNC patients, Galectin-3 correlated positively with native apical T1 (R = 0.490, *p* = 0.04) and NT-proBNP (R = 0.539, *p* = 0.02). Endothelial dysfunction biomarkers correlated positively with compact myocardium mass (R = 0.588 for ADAMTS13, *p* = 0.01; and R = 0.824 for the ADAMTS13/vWF ratio, *p* < 0.001). These findings indicate that endothelial dysfunction is greater when compacted myocardium mass decreases in LVNC patients.

## 4. Discussion

**Figure 4:**
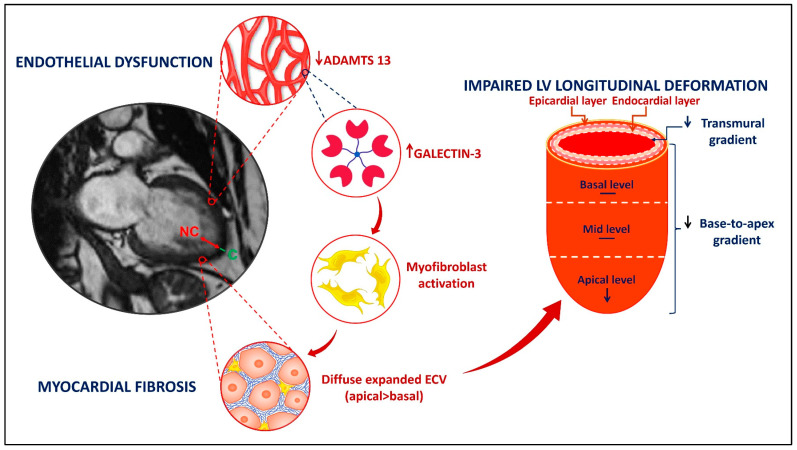
Summarizing illustration. HF in LVNC with preserved EF involves three main findings: endothelial dysfunction with a decreased level of ADAMTS13, myocardial fibrosis with an increased level of Galectin-3 leading to the activation of myofibroblasts and diffuse increase of ECV, more pronounced at the apical level of LV, and impaired LV longitudinal deformation at the apical level, leading to decrease of the transmural and base-to-apex gradients.

In this prospective study, we enrolled 21 patients with LVNC and HFpEF and compared them with 21 patients with HFpEF, but without LVNC (control group), with similar age, sex, and comorbidities. By using a multimodality approach, we showed that patients with LVNC and HFpEF have endothelial dysfunction, myocardial fibrosis, and significantly decreased longitudinal strain in the apical non-compacted LV segments, associated with increased ECV and T1 myocardial times in CMR (Figure 4). Our findings emphasize the hypothesis of a distinct form of LVNC with preserved EF, rather than an adaptative hypertrabeculation in HFpEF.

### 4.1. LVNC and Endothelial Dysfunction

This is the first study that showed lower levels of ADAMTS13 in LVNC patients, with a reduced ADAMTS13/vWF ratio, suggesting that endothelial dysfunction may play an important role in the pathogenesis of LVNC. Endothelial cells make a major contribution to ADAMTS13 production [27]; thus, levels of ADAMTS13 and the ADAMTS13/VWF ratio have been proposed as biomarkers of endothelial function [28]. In patients with LVNC, we showed positive correlations between both biomarkers of endothelial dysfunction and compact myocardium mass. Thus, as the compact myocardium mass decreases, with an increase of non-compact myocardium mass, both ADAMTS13 level and the ADAMTS13/VWF ratio decrease.

Endothelial dysfunction may also play a role in the mechanism of LVNC thromboembolic complications, since low ADAMTS13 levels were associated with an increased risk of arterial thrombosis and ischemic stroke [29].

### 4.2. LVNC and Myocardial Fibrosis

We found higher levels of Galectin-3 in patients with LVNC and HFpEF. To our knowledge, this is the first study that has evaluated the level of Galectin-3 in LVNC. Galectin-3 plays a dominant role in the fibrotic processes, being released into extracellular space, where it activates the fibroblasts [30]. It has been shown that Galectin-3 measured after STEMI is an independent predictor of increased ECV at 6 months [31]; it is also associated with myocardial replacement fibrosis assessed by LGE in patients with non-ischemic cardiomyopathy [32]. Likewise, in our study, in LVNC patients, tissue characterization by T1 mapping showed higher native apical T1 values, with diffusely expanded ECV, more extensive at the apical level, suggestive of myocardial fibrosis. Native apical T1 correlated positively with Galectin-3. NT-proBNP, higher in LVNC, also correlated positively with Galectin-3.

Our results are in accordance with previous CMR studies in LVNC [18,33] that found progressively elevated native T1 values from normal controls to LGE negative patients with LVNC, and to LGE positive patients with LVNC. Araujo-Filho et al. [18] also reported the diffuse expansion of ECV by myocardial fibrosis in LVNC patients, with both preserved or reduced LVEF, including in the myocardium without focal fibrosis (LGE-negative regions). Similar to our results, Araujo-Filho et al. [18] found the differences in native T1 and ECV, by comparison to healthy controls, mainly in the apical segments, but also in the inferior wall and septum. Szemraj-Rogucka et al. [34] demonstrated elevated levels of plasma miRNAs, which are related to fibrosis (miRNA-21, miRNA-29a, miRNA-30d, and miRNA-133a) in LVNC patients, by comparison to healthy controls, with higher values in patients with LGE as compared to patients without LGE.

Although we found diffusely expanded ECV, LGE was present in only four patients with LVNC (19%), and surprisingly in the medio-basal compacted segments. Compared to other cardiomyopathies, LGE seems to be less frequent in LVNC [35,36], and is observed in both non-compacted and compacted segments [37,38], with a higher prevalence in the compacted ones [38]. This may be explained by the fact that LGE is a marker of focal replacement fibrosis, following cardiomyocyte death in more advanced stages, and not a marker of diffuse interstitial fibrosis, which is better evaluated by T1 mapping [39] and is usually found in LVNC patients on myocardial tissue samples obtained at the time of cardiac transplantation or endomyocardial biopsy [17,40]. CMR findings of diffuse myocardial fibrosis strengthen the concept that LVNC is a diffuse process, with an early impact on the apical compact layer of non-compacted segments and a late impact on compact segments, probably related to increased wall stress caused by a progressive LV remodeling. Nucifora et al. [37] found small amounts of myocardial fibrosis in asymptomatic patients with LVNC, with preserved LVEF, suggesting that cardiac injury in LVNC might begin earlier than the onset of symptoms or LV systolic dysfunction. This observation, concordant with our data, shows that T1 mapping can be used earlier than LGE imaging to detect myocardial fibrosis in LVNC patients, from a subclinical phase. Meanwhile, Galectin-3 might be used as an early biomarker to monitor myocardial fibrosis, being more accessible than plasma miRNAs.

Mechanisms of fibrosis in LVNC are unclear and may be multifactorial. Our data showed the presence of endothelial dysfunction in LVNC patients, which can cause CMD with myocardial ischaemia, promoting diffuse fibrosis. Diminished coronary flow reserve, supported by subendocardial perfusion defects [15], has been already demonstrated in both non-compacted and compacted myocardial segments [14].

### 4.3. LVNC and Myocardial Deformation

In our study, LVNC patients had lower values of LS in the apical segments. CMR findings suggested that diffuse apical fibrosis may be the substrate of lower LS values in the apical non-compacted segments. We also found a compensatory increase in LS in basal and mid-inferior segments, with lower values of base-to-apex LS gradient. In agreement with our findings, previous studies reported reduced LS in LVNC patients with preserved EF, when compared with normal subjects [41,42]. Bellavia et al. [42] showed also that LS was more impaired in the apical segments.

LVNC is also different from other cardiomyopathies. Thus, patients with LVNC with reduced EF showed lower apical LS values than patients with HCM, with a reduced base-to-apex deformation gradient [43]. By comparison with DCM, patients with LVNC had higher apical LS values, with an increased base-to-apex deformation gradient [3]. In fact, Tarando et al. [44] reported that the base-to-apex deformation gradient is the best parameter to distinguish LVNC from DCM [44].

In LVNC, trabeculations are commonly localized in the endocardial layers, as the myocardial compaction progresses from epicardium to endocardium. By assessing multilayer LS, we found lower values of LS in the endocardial layers, with significantly lower transmural LS.

### 4.4. Clinical Implications

Our findings suggest that subclinical myocardial fibrosis is the substrate of decreased apical myocardial deformation. Subclinical myocardial fibrosis might be monitored by Galectin-3. Thus, reducing Galectin-3 level might be a new therapeutical target to prevent myocardial fibrosis. Furthermore, if endothelial dysfunction is validated in future large-scale studies, ADAMTS13 level and the ADAMTS13/VWF ratio might be used in clinical practice to monitor the progression of endothelial dysfunction, and these biomarkers might become other therapeutical targets in patients with LVNC. Finally, the assessment of longitudinal strain by speckle tracking in patients with LVNC should become mandatory for diagnosis and for monitoring treatment.

### 4.5. Limitations

There are several limitations of our study. First, this is a single-center study with a rather limited number of patients. However, LVNC is a rare disease, and we included symptomatic patients with LVNC and HFpEF, carefully diagnosed by strict criteria, and compared them with matched patients with HFpEF. Considering the reduced number of patients, this study is hypothesis-generating research that needs validation in a large-scale study, ideally by an international multicenter collaboration. Second, there is a limited number of men in both groups, due to the mandatory presence of a diagnosis of HFpEF (by protocol), which is more frequent in women than men. Third, asymptomatic coronary artery disease was not ruled out by either coronary angiography or computed tomography. Finally, genetic testing was unavailable; we consider that it would have been important to corelate our findings with a genetic diagnosis.

## 5. Conclusions

Patients with LVNC and HFpEF had endothelial dysfunction, expressed by a lower ADAMTS13 level and ADAMTS13/vWF ratio, and the overexpression of Galectin-3. These lead to diffuse myocardial fibrosis, more extensive at the apical level, explaining the decrease in apical deformation. The positive correlation of NC myocardium with ECV base-to-apex gradient, together with the decrease in transmural and base-to-apex deformation gradients, reinforces the hypothesis of myocardial maturation failure and its sequence. All these findings support the idea that LVNC with HFpEF may be a distinct phenotype, rather than an adaptative hypertrabeculation process.

## Figures and Tables

**Table 1:** Characteristics of the study groups.

	LVNC (*n* = 21)	Control (*n* = 21)	*p*-Value
Clinical characteristics			
Age (years)	61.5 ± 8.7	66.0 ± 6.3	0.06
Female gender (%)	81.0	85.7	0.67
BMI (kg/m^2^)	30.2 ± 5.7	29.3 ± 3.9	0.55
Heart rate (bpm)	66.6 ± 10.3	66.0 ± 10.8	0.85
Systolic blood pressure (mmHg)	142.7 ± 17.3	138.1 ± 22.5	0.46
Smoking (%)	23.8	14.3	0.43
Obesity (%)	47.6	42.9	0.76
Hypertension (%)	90.5	95.2	0.54
Dyslipidaemia (%)	90.5	85.7	0.63
Diabetes mellitus (%)	28.6	33.3	0.73
CKD ^#^ (%)	4.8	9.5	0.55
History of AFib (%)	9.5	14.3	0.64
LV volumes and systolic function parameters			
LVEDVi_CMR_ (mL/m^2^)	77.8 ± 12.8	70.2 ± 10.9	0.04
LVESVi_CMR_ (mL/m^2^)	30.9 ± 9.0	26.7 ± 5.3	0.07
LVEF_CMR_ (%)	60.8 ± 6.5	62.3 ± 5.3	0.40
LVEF_TTE_ (%)	59.7 ± 5.2	60.3 ± 4.7	0.69
LS	−19.3 ± 2.7	−20.2 ± 2.2	0.25
Structural and diastolic function parameters			
LVMI (g/m^2^)	101.4 ± 21.8	103.1 ± 30.2	0.84
Relative wall thickness	0.46 ± 0.07	0.50 ± 0.06	0.10
LAVI (mL/m^2^) *	39.7 (15.2)	39.5 (8.7)	0.97
PASP (mmHg) *	34.0 (5.5)	33.0 (9.0)	0.40
E/e’ ratio	10.2 ± 3.8	11.4 ± 2.7	0.26
Biomarkers			
NT-proBNP (pg/mL) *	237 (156–489)	156 (139–257)	0.04
Galectin-3 (ng/mL) *	7.3 (6.0–11.5)	5.6 (4.8–8.3)	0.04
ADAMTS13 (ng/mL)	767.3 ± 335.5	962.3 ± 253.7	0.04
vWF (ng/mL) *	25.2 (23.1–30.1)	24.0 (21.4–26.3)	0.16
ADAMTS13/vWF ratio *	31.3 (14.8–42.3)	40.8 (32.0–52.5)	0.03

Continuous variables are presented as mean ± standard deviation or median (IQR) (*) and categorical variables as frequencies (%). ADAMTS13, a disintegrin and metalloproteinase with thrombospondin type 1 motif-13; AFib, atrial fibrillation; BMI, body mass index; CKD, chronic kidney disease (^#^ estimated glomerular filtration rate between 45–59 mL/min/1.73 m^2^); CMR, cardiac magnetic resonance; LVEDVi, left ventricular end-diastolic volume index; LVESVi, left ventricular end-systolic volume index; LVEF, left ventricular ejection fraction; LVMI, left ventricular mass index; LAVI, left atrial volume index; LS, longitudinal strain; NT-proBNP, N-terminal pro-B-type natriuretic peptide; PASP, pulmonary artery systolic pressure; TTE, transthoracic echocardiography; vWF, von Willebrand factor.

**Table 2:** Comparison between groups for cardiac magnetic resonance parameters.

Parameter	LVNC (*n* = 21)	Control (*n* = 21)	*p*-Value
Native T1			
T1 global (ms)	1014 ± 32	1003 ± 28	0.26
T1 basal (ms)	1003 ± 27	1004 ± 29	0.82
T1 mid (ms)	997 ± 36	999 ± 31	0.82
T1 apical (ms)	1061 ± 72	1008 ± 40	0.005
T1 base-to-apex gradient (ms) *	41 (23–86)	2.5 (−28–28)	0.002
ECV			
ECV global (%)	27.2 ± 2.9	24.4 ± 2.5	0.002
ECV basal (%)	26.2 ± 2.9	24.3 ± 2.6	0.03
ECV mid (%)	26.6 ± 3.3	23.8 ± 2.6	0.005
ECV apical (%)	29.6 ± 3.8	25.2 ± 2.8	<0.001
ECV base-to-apex gradient (%) *	2.8 (1.2–5.6)	0.9 (0.1–2.1)	0.01
ECV by segments			
Segment 1: basal anterior (%)	25.1 ± 3.3	23.0 ± 3.0	0.03
Segment 2: basal anteroseptal (%)	26.4 ± 3.5	25.7 ± 3.7	0.50
Segment 3: basal inferoseptal (%)	26.7 ± 4.1	23.6 ± 2.4	0.005
Segment 4: basal inferior (%)	26.4 ± 3.7	23.8 ± 3.1	0.02
Segment 5: basal inferolateral (%)	26.3 ± 3.4	25.2 ± 3.8	0.31
Segment 6: basal anterolateral (%)	26.1 ± 4.3	24.3 ± 3.6	0.17
Segment 7: mid-anterior (%)	25.9 ± 3.7	23.4 ± 2.6	0.01
Segment 8: mid-anteroseptal (%)	26.5 ± 3.1	24.0 ± 2.8	0.01
Segment 9: mid-inferoseptal (%)	26.7 ± 3.7	23.9 ± 2.6	0.01
Segment 10: mid-inferior (%)	25.9 ± 4.2	23.0 ± 2.9	0.01
Segment 11: mid-inferolateral (%)	27.1 ± 5.6	24.5 ± 3.4	0.07
Segment 12: mid-anterolateral (%)	27.6 ± 4.3	24.1 ± 4.0	0.01
Segment 13: apical anterior (%)	30.5 ± 4.4	25.2 ± 2.9	<0.001
Segment 14: apical septal (%)	29.0 ± 3.7	24.9 ± 2.8	<0.001
Segment 15: apical inferior (%)	28.7 ± 4.8	24.7 ± 3.4	0.004
Segment 16: apical lateral (%)	30.2 ± 5.2	25.9 ± 3.3	0.004

Continuous variables are presented as mean ± standard deviation or median and IQR (*).

**Table 3:** Comparison between groups for longitudinal strain by levels, layers, and segments, assessed by speckle tracking echocardiography.

Parameter	LVNC (*n* = 21)	Control (*n* = 21)	*p*-Value
Strain by levels			
LS basal (%)	−17.5 ± 2.6	−17.3 ± 2.4	0.82
LS mid (%)	−18.9 ± 2.6	−18.7 ± 2.5	0.79
LS apical (%)	−21.4 ± 4.4	−24.3 ± 3.2	0.01
LS base-to-apex gradient (%)	3.8 ± 4.7	6.9 ± 3.4	0.02
Strain by layers			
LS endo (%)	−21.4 ± 2.7	−22.7 ± 2.6	0.11
LS epi (%)	−17.4 ± 2.5	−17.9 ± 1.9	0.50
LS transmural gradient (%)	3.9 ± 0.8	4.8 ± 1.0	0.006
Strain by segments			
Segment 1: basal anterior (%)	−16.0 ± 3.9	−18.1 ± 3.6	0.07
Segment 2: basal anteroseptal (%)	−16.4 ± 5.4	−15.6 ± 3.1	0.58
Segment 3: basal inferoseptal (%)	−13.9 ± 3.1	−13.7 ± 3.4	0.85
Segment 4: basal inferior (%)	−20.1 ± 4.4	−17.4 ± 3.2	0.03
Segment 5: basal inferolateral (%)	−20.5 ± 5.0	−19.5 ± 4.0	0.48
Segment 6: basal anterolateral (%)	−18.2 ± 4.0	−19.2 ± 3.1	0.19
Segment 7: mid-anterior (%)	−16.1 ± 4.3	−17.5 ± 3.4	0.27
Segment 8: mid-anteroseptal (%)	−20.9 ± 3.7	−20.5 ± 4.3	0.79
Segment 9: mid-inferoseptal (%)	−17.9 ± 3.1	−18.4 ± 3.4	0.61
Segment 10: mid-inferior (%)	−21.8 ± 3.6	−19.4 ± 3.2	0.03
Segment 11: mid-inferolateral (%)	−18.8 ± 3.3	−18.6 ± 3.9	0.90
Segment 12: mid-anterolateral (%)	−18.1 ± 4.1	−17.8 ± 4.3	0.85
Segment 13: apical anterior (%)	−20.3 ± 6.0	−23.8 ± 4.6	0.04
Segment 14: apical septal (%)	−22.3 ± 3.7	−26.0 ± 3.3	0.002
Segment 15: apical inferior (%)	−23.2 ± 5.8	−25.2 ± 3.4	0.19
Segment 16: apical lateral (%)	−19.6 ± 4.3	−22.5 ± 4.0	0.03
Segment 17: apex (%)	−21.4 ± 4.5	−24.2 ± 3.1	0.02

Variables are presented as mean ± standard deviation.

## Data Availability

The data presented in this study are available on request from the corresponding author.
